# Atsttrin Promotes Cartilage Repair Primarily Through TNFR2-Akt Pathway

**DOI:** 10.3389/fcell.2020.577572

**Published:** 2020-10-29

**Authors:** Jianlu Wei, Kaidi Wang, Aubryanna Hettinghouse, Chuanju Liu

**Affiliations:** ^1^Department of Orthopaedic Surgery, Qilu Hospital of Shandong University, Jinan, China; ^2^Department of Orthopaedic Surgery, New York University Langone Medical Center, New York, NY, United States; ^3^Department of Cell Biology, New York University Grossman School of Medicine, New York, NY, United States

**Keywords:** Atsttrin, chondrogenesis, cartilage repair, TNFR2, signaling

## Abstract

**Background:**

Cartilage defects account for substantial economic and humanistic burdens and pose a significant clinical problem. The efficacy of clinical approaches to cartilage repair is often inadequate, in part, owing to the restricted proliferative capacity of chondrocytes. Molecules have the capacity to promote the differentiation of multipotent mesenchymal stem cells into chondrocytes and may also gain the ability to repair the damaged cartilage.

**Objective:**

This study aimed to investigate the role of Atsttrin (progranulin-derived engineered protein) in cartilage repair as well as the signaling pathway involved.

**Methods:**

Primary and mesenchymal stem cell lines were used for the micromass culture. A murine cartilage defect model was used to determine the role of Atsttrin in cartilage repair *in vivo*. Real-time polymerase chain reaction and Western blot analysis were used to monitor the effect of Atsttrin on the transcriptional and protein levels, respectively, of key anabolic and catabolic signaling molecules.

**Results:**

Atsttrin stimulated chondrogenesis *in vitro* and accelerated cartilage repair *in vivo.* In addition, Atsttrin-mediated cartilage repair occurred primarily through tumor necrosis factor receptor 2-initiated Akt signaling and downstream JunB transcription factor.

**Conclusion:**

Atsttrin might serve as a promising therapeutic modality for cartilage regeneration.

## Introduction

Articular cartilage diseases affect more than 273 million of adults across the world (Helmick et al., 2008; Lawrence et al., 2008; Krishnan and Grodzinsky, 2018). Damage to the articular cartilage can result in potentially crippling symptoms, such as swelling, pain and decreased mobility, and, if left untreated, osteoarthritis (OA). Suboptimal therapeutic outcomes may be largely attributable to the limited reparative competence of chondrocytes (Berthiaume et al., 2011). Current surgical treatments include joint replacement, osteotomies, microfracture, autologous chondrocyte implantation, and/or allografts and autografts (Detterline et al., 2005; Devitt et al., 2017). Even minor cartilage injuries may lead to persistent tissue damage and eventual OA. Improved corrective approaches for cartilage damage are critical to limiting humanistic and financial losses incurred following cartilage injury.

Progranulin (PGRN) is a growth factor–like molecule with multiple functions in diverse biological processes (Wei et al., 2016; Cui et al., 2019). A previous study reported that PGRN was expressed in human articular cartilage. The level of PGRN was significantly upregulated in diseased cartilage with osteoarthritis (OA) and rheumatoid arthritis (Zhao et al., 2015). Additionally, PGRN is important for chondrocyte proliferation and differentiation, which are functions recapitulated in animal models of cartilage defect (Feng et al., 2010; Kong et al., 2016). Recent studies reported that PGRN and its engineered derivative Atsttrin demonstrated a therapeutic effect in inflammatory and degenerative arthritis murine models through binding to tumor necrosis factor receptors (TNFRs) (Tang et al., 2011; Wei et al., 2017; Wei and Liu, 2018). Atsttrin, comprising half-units of granulins A, C, and F plus linkers P3, P4, and P5, lacks the oncogenic activity of PGRN. However, Atsttrin has a longer half-life (about 120 h) compared with PRGN (about 40 h) (Tang et al., 2011; Liu et al., 2014).

Considering the stimulatory role of PGRN in chondrogenesis and the protective effects of PGRN-derived Atsttrin in animal models of arthritis (Feng et al., 2010; Tang et al., 2011; Xia et al., 2015; Wei et al., 2017), it was hypothesized that Atsttrin might represent a novel potential treatment for cartilage regeneration. This study found that Atsttrin could promote chondrogenesis *in vitro* and accelerated cartilage repair in a mouse full-thickness cartilage defect model wherein the subchondral bone was penetrated to allow for an influx of marrow-derived mesenchymal stem cells (MSCs). Mechanistic studies demonstrated that Atsttrin-mediated cartilage repair occurred primarily through TNFR2 signaling.

## Materials and Methods

### Media and Reagents

Dulbecco’s modified Eagle Medium (DMEM) and fetal bovine serum were purchased from Gibco-BRL (Sydney, Australia). Specific antibodies against JunB (Cat. #sc-73) and glyceraldehyde-3-phosphate dehydrogenase (GAPDH, Cat. # sc-25778) were obtained from Santa Cruz Biotechnology, Inc. (TX, United States). Phosphatidylinositol 3-kinase (PI3K), Akt, and MAPK/ERK1/2 activation were assessed using PathScan Multiplex Western Cocktail I from Cell Signaling (Cat. #5301, MA, United States). Secondary horseradish peroxidase–conjugated antibody was purchased from Jackson Immunoresearch Inc. (Cat. # 711-035-152, PA, United States). The blots were developed using Western Lightning Plus-ECL from Perkin-Elmer (Cat. # NEL103001EA, MA, United States). Tris, glycine, sodium dodecyl sulfate (SDS), and other regents were obtained from Sigma (MO, United States) unless stated otherwise.

### Effect and Mechanism of Atsttrin on Chondrogenesis

Bone marrow stem cells (BMSCs) were obtained from mice, and multipotential murine C3H10T1/2 cells were also used for this experiment. Chondrogenic differentiation medium, consisted of high-glucose (4.5 g/L) DMEM supplemented with ITS + (Collaborative Research, MA, United States), 0.1 μM dexamethasone, and 50 μg/mL ascorbate 2-phosphate with 100 ng/mL BMP2 or 1000 ng/mL Atsttrin, was used for differentiation. Micromass culture was used for the induction of chondrogenesis. In detail, 500,000 BMSCs at passage 2 were plated in the center of a culture plate, and the cells were incubated at 37°C with 5% CO_2_ for 3 h. After cell aggregation, the chondrogenic medium was carefully added to the cells. BMSCs isolated from wild-type (WT), TNFR1-deficient (TNFR1–/–), and TNFR2-deficient (TNFR2–/–) mice underwent chondrogenic induction for 10 days prior to safranin O staining and Alcian blue staining to determine chondrocyte differentiation.

The aforementioned micromass culture of C3H10T1/2 cells was performed in the absence or presence of Atsttrin at a concentration of 1000 ng/mL to examine the effects of Akt-signaling blockade on Atsttrin-mediated chondrogenesis. After 5 days in culture, 0.01% DMSO (*v*/*v*) or 1 μM Wortmannin was added to the cultured cells and further incubated for 2 or 7 days prior to collection for real-time polymerase chain reaction (rtPCR) analysis. C3H10T1/2 cells were stably transfected with pSuper vector, pSuper-JunB expressing a small interfering RNA (siRNA) against JunB (Feng et al., 2010), pCMV-JunB expression plasmid or sequential knockdown and expression plasmids using *Lipofectamine 2000* in serum-free medium with 6-h incubation, following the manufacturer’s protocols, to examine the importance of downstream JunB transcription factor activation in Atsttrin-mediated chondrogenesis. A fresh complete medium was added, and the transfected cells were cultured in micromass with or without 1000 ng/mL Atsttrin for 2 or 7 days for the induction of chondrogenesis prior to collection for rtPCR.

### Real-Time PCR Assay

Micromass cultures of C3H10T1/2 cells and BMSCs were treated with commercially available recombinant BMP2 prior to RNA extraction. The cells were harvested from 3 wells of 12-well plates, and 1 μg of total RNA per sample was reverse-transcribed using the Promega ImProm-II Reverse Transcription System (WI, United States). Quantitative real-time PCR was performed using the following sequence-specific primers: 5′-TGGTGGAGCAGCAAGAGCAA-3′ and 5′-CAGTGGACAGTA GACGGAGGAAA-3′ for collagen type II alpha 1 (*Col2a1*); 5′-CC TGCTACTTCATCGACCCC-3′ and 5′-AGATGCTGTTGACTC GAACCT-3′ for Aggrecan (*Acan*); 5′-GAGGCCACGGAA CAGACTCA-3′ and 5′-CAGCGCCTTGAAGATACGATT-3′ for Sox9; and 5′-AGGTCGGTGTGAACGGATTTG-3′ and 5′-TGTAGACCATGTAGTTGAGGTCA-3′ for *Gapdh*. The reactions were carried out in an *Applied Biosystems 7300* Sequence Detection System (CA, United States). In detail, more than 40 cycles of 15 s at 95°C and 1 min at 60°C were used for the experiment. GAPDH was employed as an internal control. Each sample and gene were evaluated in triplicate.

### Murine Osteochondral Defect Model

All animal studies were performed following the institutional guidelines, and all performances were approved by the Institutional Animal Care and Use Committee of New York University. The mice were group-housed within the rodent barrier facility at the Skirball Institute of Biomolecular Medicine with a standard assessment of food and water. The animal room had a specific-pathogen-free environment. The temperature and humidity were automatically controlled in accordance with a 12-h light/dark cycle. C57BL6/J background WT, TNFR1 knockout (TNFR1–/–), and TNFR2 knockout (TNFR2–/–) mice were acquired from Jackson Laboratory and maintained within the animal housing facility. The genotyping and housing of TNFR1–/–, TNFR2–/–, and WT littermate mice was performed as described previously (Tang et al., 2011). Eight-week-old male mice were used for this study.

The model was established and modified as previously described (Matsuoka et al., 2015). Briefly, the animals were subjected to general anesthesia. After that, the hind limbs were sterilized, and an ophthalmic ointment was applied to the eyes prior to positioning to a surgical microscope (SZX16; Olympus, Tokyo, Japan). A microsurgical scalpel was used for the anterior approach. The skin and the joint capsule were gently opened, and the patella was dislocated for exposing the trochlear groove. A unilateral, longitudinal full-thickness injury was generated along the articular surface of the trochlear groove using a constructed device comprising a 27G needle sheathed in a bisected 21G needle; the 21G needle was adjusted to expose 300 mm of the 27G needle beveled end. Subchondral bleeding was taken as indicative of the successful generation of the defect. The surgical site was irrigated with sterile saline, and a collagen sponge containing phosphate-buffered saline (PBS) (*n* = 6) or 6 μg Atsttrin (*n* = 6) was inserted into the defect site prior to stepwise suturing of the joint capsule and skin. The mice were postoperatively monitored for anesthetic recovery with thermal support. The contralateral limbs were subjected to a sham procedure wherein no defect was generated. The mice were sacrificed 6 weeks after the surgery.

### Histological Analysis

The harvested knee joint tissues were fixed with 4% PFA for 2 days at room temperature. The tissues were then decalcified for 2 weeks in 10% EDTA (*w*/*v*) on a shaker at 4°C before dehydration, paraffin pre-processing, and embedding. Further, 5-μm serial sections were cut and stained with safranin O/fast green for the evaluation of cartilage repair. The degree of repair was evaluated by a blinded investigator using an International Cartilage Repair Score (ICRS) for cartilage repair (Mainil-Varlet et al., 2010).

### Western Blot Analysis

The micromass cultures of BMSCs underwent starvation for 24 h to determine Atsttrin-mediated signaling in chondrogenesis. After that, the cells were stimulated with 1000 ng/mL Atsttrin over varied time courses, and the whole-cell lysates were collected for Western blot analysis. The cells were harvested and mixed with 5 × sample buffer (312.5 mM Tris-HCl, pH 6.8; 5% β-mercaptoethanol; 10% SDS; 0.5% bromphenol blue; and 50% glycerol). The samples were boiled at 95°C for 5 min, allowed to cool, and resolved on a 10% SDS-polyacrylamide gel followed by electro-transferring. After the blockage with 5% non-fat milk, the blots on the membrane were incubated overnight for 1 h with primary antibodies at the manufacturer-indicated assay-dependent dilution factor. BMSCs were starved for 24 h, followed by the stimulation with 1000 ng/mL Atsttrin over various time courses, to examine Atsttrin-mediated signaling in chrondrogenesis. The cells were collected, and the lysates were incubated with the PathScan Multiplex Western Cocktail I at 1:200 dilution. The cell lysates were incubated with a rabbit anti-JunB polyclonal antibody (1:1000 dilution) to determine the induction of JunB by Atsttrin. The blots were subjected to three 5-min washes with TBST prior to 1-h incubation with the horseradish peroxidase–conjugated anti-rabbit secondary antibody (1:2000 dilution), repeated washing, and signal development with Western Lightning Plus-ECL.

### Statistical Analysis

The results were expressed as mean values ± standard error of the mean. Statistical significance was assessed using ANOVA and Student’s *t* test with SPSS software (SPSS Inc, IL, United States). The data were checked for normality before analysis. A *P*-value < 0.05 indicated a statistically significant difference.

## Results

### Atsttrin Stimulated Chondrogenesis *in vitro*

**FIGURE 1:**
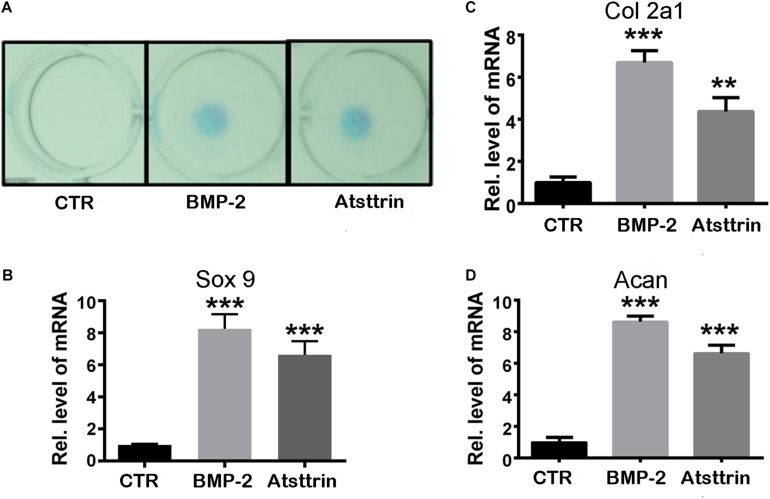
Atsttrin promoted chondrogenesis in C3H10T1/2 cells. **(A)** C3H10T1/2 cells were incubated in the absence (CTR) or presence of 100 ng/mL BMP2 or 1000 ng/mL Atsttrin for 10 days, followed by Alcian blue staining. **(B–D)** C3H10T1/2 cells were stimulated with either 100 ng/mL BMP2 or 1000 ng/mL Atsttrin, and real-time PCR was performed to examine the expression of Sox9, collagen II (Col2a1), and aggrecan (Acan). Units were arbitrary; normalized values were calibrated against controls and set as 1. Experiments were conducted in triplicate. Three independent experiments were performed. ***P < 0.001, **P < 0.01.

**FIGURE 2:**
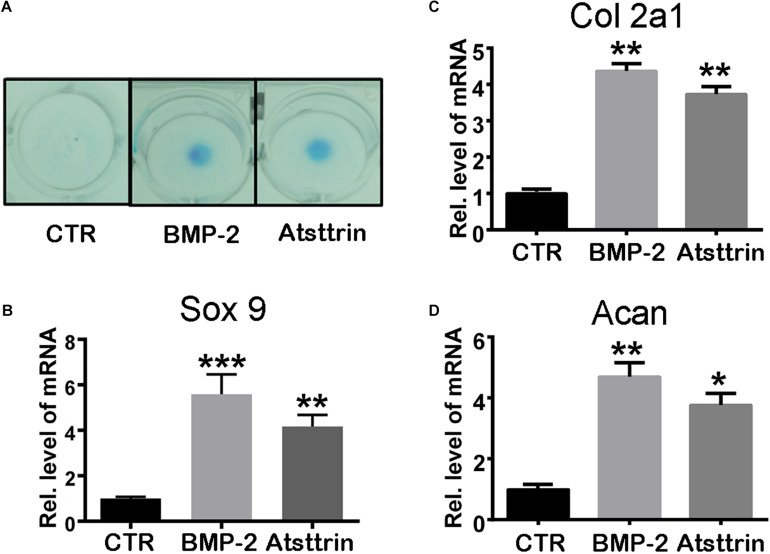
Atsttrin promoted chondrogenesis in mouse primary bone marrow stem cells (BMSCs). **(A)** BMSCs were incubated in the absence (CTR) or presence of 100 ng/mL BMP2 or 1000 ng/mL Atsttrin for 10 days, followed by Alcian blue staining. **(B–D)** BMSCs were stimulated with either 100 ng/mL BMP2 or 1000 ng/mL Atsttrin, and real-time PCR was performed to examine the expressions of Sox9, collagen II (Col2a1), and aggrecan (Acan). Values were normalized to controls and set as 1. Experiments were conducted in triplicate. Three independent experiments were performed. ***P < 0.001, **P < 0.01, *P < 0.05.

Given the stimulatory effect of PGRN on chondrogenesis, the present study first sought to investigate whether PGRN-derivative Atsttrin could similarly induce chondrogenesis. The chondrogenic potential of Atsttrin and BMP-2, a growth factor and well-known inducer of chondrogenic differentiation, were compared. A pluripotent stem cell line C3H10T1/2 capable of differentiation into chondrocytes was employed for these assays (Denker et al., 1999). The micromass cultured cells were incubated in the presence of 1000 ng/mL Atsttrin or 100 ng/mL BMP-2 for 10 days. As shown in Figure 1A, Alcian blue staining demonstrated chondrocyte differentiation in both the BMP-2- and Atsttrin-treated groups. Chondrogenesis was also examined at the transcriptional level by testing the expression of marker genes specific for chondrocytes. As shown in Figures 1B–D, BMP-2 and Atsttrin significantly induced the expression of Sox 9, collagen II, and aggrecan. BMSCs were maintained in a micromass culture system as described earlier for 10 days. As shown in Figure 2A, Alcian blue staining validated the occurrence of Atsttrin-stimulated chondrogenesis in primary cells. Real-time PCR revealed significant upregulation of the expression of the chondrogenic marker genes, Sox9, collagen II, and aggrecan, in the treatment groups (Figures 2B–D). Collectively, these results indicated that Atsttrin could induce chondrocyte differentiation *in vitro*.

### Atsttrin Promoted Cartilage Repair *in vivo* Primarily Through TNFR2

**FIGURE 3:**
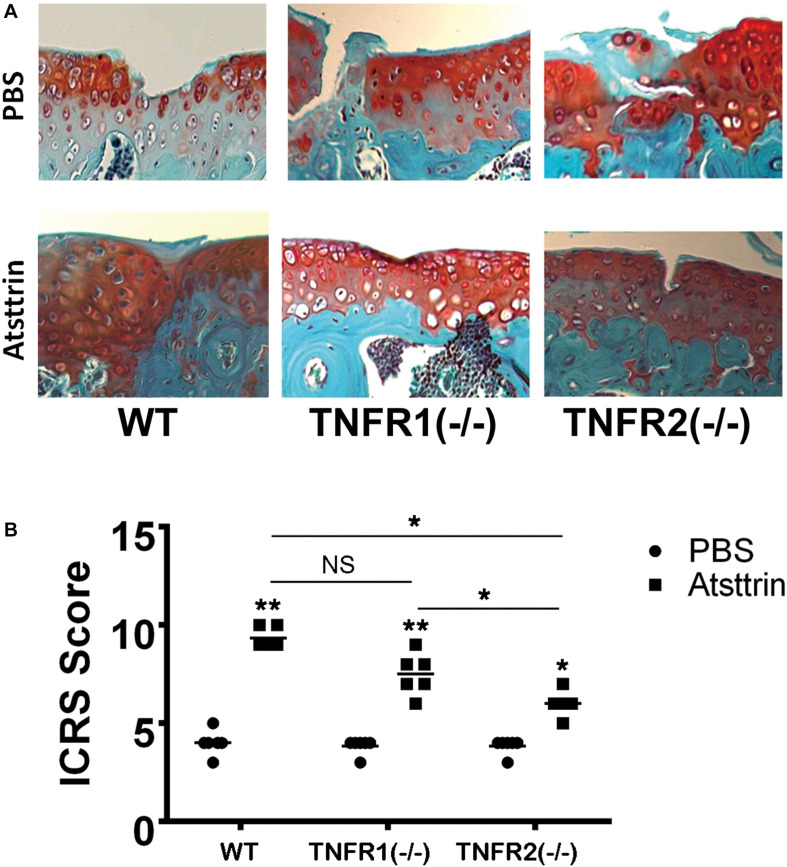
Atsttrin accelerated cartilage repair through TNFRs. **(A)** Atsttrin promoted cartilage regeneration following surgically induced cartilage defect model relative to PBS (*n* = 6 per group), assayed using safranin O staining. **(B)** International Cartilage Repair Score (ICRS) based on the result of safranin O staining.

Considering a positive effect of Atsttrin on *in vitro* chondrogenesis, the study determined whether Atsttrin could accelerate cartilage regeneration *in vivo*. Atsttrin is composed of three TNFR-binding fragments (1/2F-1/2A-1/2C) of PGRN and exhibits selective TNFR-binding ability (Tang et al., 2011; Tian et al., 2014). Articular cartilage defects were established in the femoral trochlea of WT, TNFR1–/–, and TNFR2–/– mice, and a collagen sponge loaded with PBS or 6 μg Atsttrin was intra-operatively administered to investigate whether TNFR1 or TNFR2 or both receptors mediated the effect of Atsttrin on chondrogenesis and cartilage repair *in vivo*. The mice were sacrificed after 6 weeks for *ex vivo* evaluation. As indicated in Figure 3 and Supplementary Figure 1, the scoring of Safranin O/Fast green staining indicated that Atsttrin could promote cartilage repair in WT, TNFR1–/–, and TNFR2–/– mice relative to their PBS-treated counterparts. Atsttrin-mediated cartilage repair showed no difference between TNFR1–/– mice and WT mice. However, Atsttrin-mediated cartilage repair was largely reduced in TNFR2–/– mice compared with WT mice. Additionally, Atsttrin-mediated cartilage repair was significantly reduced in TNFR2–/– mice compared with TNFR1–/– mice. These results indicated that Atsttrin-mediated cartilage repair depended mainly on the presence of TNFR2.

### Atsttrin Promoted Cartilage Repair Through TNFR2-Akt Signaling

**FIGURE 4:**
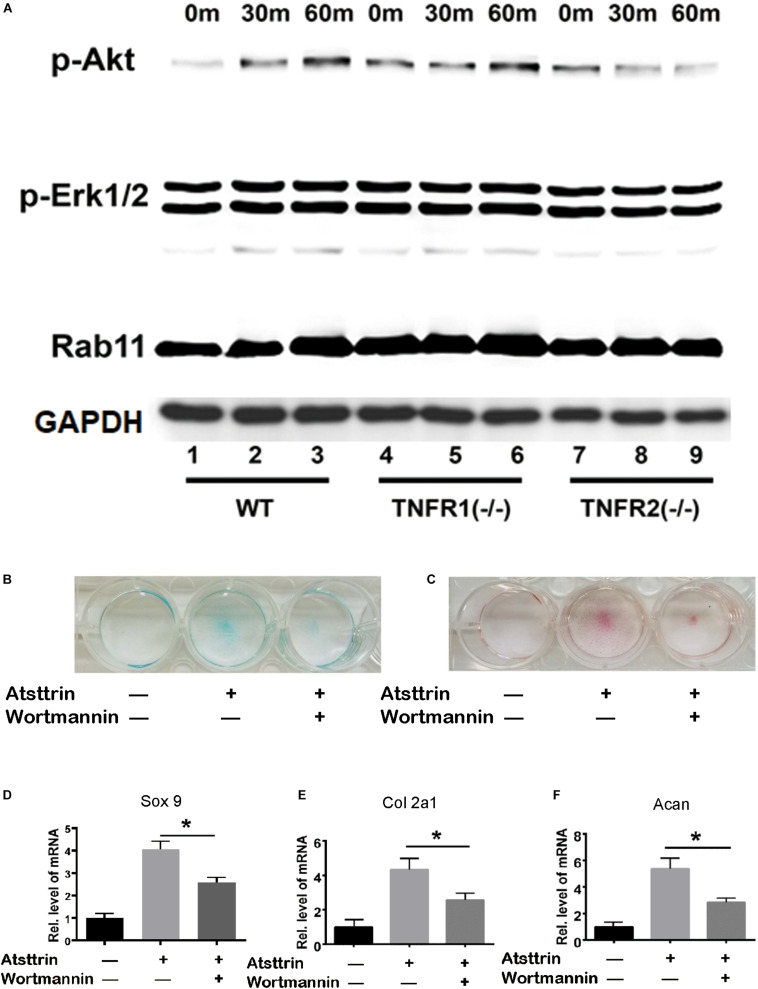
Atsttrin-mediated chondrogenesis depended on TNFR2-Akt signaling. **(A)** Primary bone marrow stem cells were isolated from WT, TNFR1–/–, and TNFR2–/– mice, cultured in the presence of 1000 ng/mL Atsttrin, and collected at various time points, followed by Western blot analysis using PathScan Multiplex Western Cocktail I. **(B,C)** BMSCs were incubated in the absence (CTR) or presence of 1000 ng/mL Atsttrin with or without 1 μM Wortmannin for 10 days, followed by Alcian blue staining and safranin O staining. **(D–F)** BMSCs were incubated in the absence (CTR) or presence of 1000 ng/mL Atsttrin with or without 1 μM Wortmannin, and the expression of Sox9, collagen II (Col2a1), and aggrecan (Acan) was measured using real-time PCR. Values were normalized to controls, here given the value of 1. Three independent experiments were performed. *P < 0.05.

BMSCs were isolated from WT, TNFR1–/–, and TNFR2–/– mice and subjected to chondrogenic differentiation prior to treatment with Atsttrin to investigate the signaling pathways involved. The cells were collected for Western blot analysis of signaling pathway activation using an antibody cocktail. As illustrated in Figure 4A, Atsttrin treatment did not activate Erk1/2 signaling in WT, TNFR1–/–, or TNFR2–/– BMSCs. Atsttrin activated Akt signaling in both WT and TNFR1–/– BMSCs, while activation was nearly abolished in TNFR2–/– chondrocytes.

BMSCs were incubated in the chondrogenic medium in the absence (CTR) or presence of 1000 ng/mL Atsttrin with or without PI3K/Akt-signaling inhibition using 1μM Wortmannin for 10 days, followed by Safranin O staining and Alcian blue staining. As indicated in Figures 4B,C, Atsttrin effectively promoted chondrogenesis in the absence of Wortmannin; however, the inclusion of Wortmannin remarkably reduced chondrocyte differentiation. Moreover, the transcriptional levels of Sox 9, aggrecan, and collagen II significantly increased in presence of Atsttrin and decreased in the presence of Wortmannin relative to the levels in control cells (Figures 4D–F). These results indicated that TNFR2/Akt signaling was integral to the chondrogenic effect of Atsttrin.

### Jun B Was a Downstream Molecule of Atsttrin-Mediated Chondrogenesis

**FIGURE 5:**
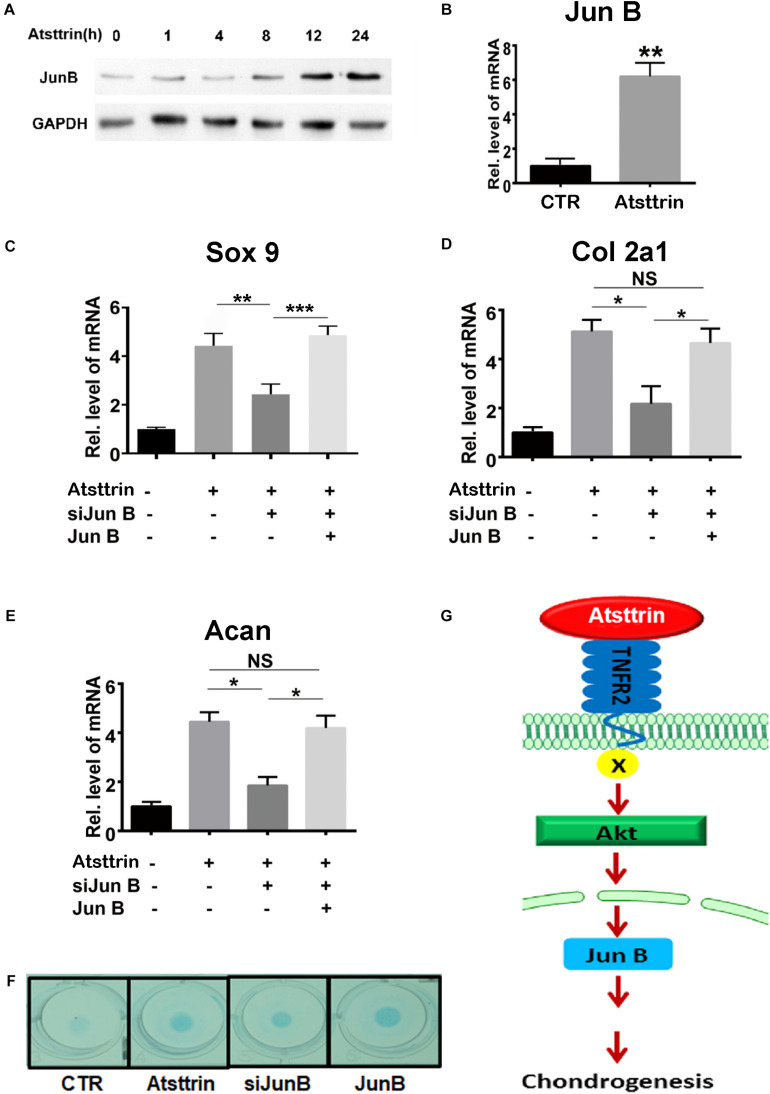
Jun B was a downstream molecule of Atsttrin-mediated chondrogenesis. **(A)** Protein expression of Jun B in the presence of Atsttrin. C3H10T1/2 cells were micromass cultured with 1000 ng/mL Atsttrin for various time points, followed by Western blot analysis. **(B)** Transcriptional level of Jun B in C3H10T1/2 cells. C3H10T1/2 cells were micromass cultured in the absence or presence of 1000 ng/mL Atsttrin for 6 h, followed by real-time PCR. **(C–E)** Expression of Sox9, collagen II (Col2a1), and aggrecan (Acan) in C3H10T1/2 cells, which were micromass cultured in the absence or presence of 1000 ng/mL Atsttrin with or without transfection. C3H10T1/2 cells were transfected with pSuper JunB encoding an siRNA interfering with JunB expression, or a plasmid expressing JunB, or each plasmid in combination. Values were normalized to controls, here given the value of 1. Three independent experiments were performed. *P < 0.05. **(F)** C3H10T1/2 cells were incubated in the absence (CTR) or presence of 1000 ng/mL Atsttrin with transfected SiJunB or JunB for 10 days, followed by Alcian blue staining. **(G)** A proposed model for the role of Atsttrin in chondrogenesis.

A previous study demonstrated that PGRN promoted chondrogenesis, at least in part, through the JunB transcription factor acting as a critical downstream mediator of chondrocyte differentiation (Feng et al., 2010). C3H10T1/2 cells were maintained in the micromass culture in the presence or absence of 1 μg/mL Atsttrin for various time points prior to protein or RNA collection for Western blot analysis and rtPCR, respectively, to address the potential existence of JunB as a shared downstream target of PGRN and Atsttrin. As shown in Figure 5A, the protein expression of JunB increased in Atsttrin-treated cells beginning at the 12-h treatment time point. As shown in Figure 5B, the transcriptional level of JunB also significantly increased following 6-h culture with Atsttrin. The study next determined whether silencing JunB could inhibit Atsttrin-mediated chondrogenesis by transfecting C3H10T1/2 cells with pSuper JunB encoding an siRNA (siJunB), or a plasmid overexpressing JunB, or each plasmid in combination. RNA was collected for PCR following the micromass culture in the absence or presence of Atsttrin. As indicated in Figures 5C–E, the transcriptional expression of Sox9, collagen II, and aggrecan significantly increased in the presence of Atsttrin. In contrast, siJunB remarkably reduced their expression. Additionally, the Atsttrin-mediated expression of Sox9, collagen II, and aggrecan were restored after JunB expression was restored. In addition, Alcian blue staining of micromasses of transfected cells with JunB overexpression and siRNA against JunB also demonstrated that JunB was required for Atsttrin-stimulated chondrogenesis (Figure 5F). Taken together, these results indicated that Atsttrin-mediated chondrogenesis, similar to previous observations of PGRN-stimulated chondrogenesis (Feng et al., 2010), depended on the activity of the JunB transcription factor.

## Discussion

The preset study examined the chondrogenic potential and underlying mechanism of PGRN-derivative Atsttrin. It was hypothesized that Atsttrin would promote chondrogenic differentiation and accelerate cartilage repair through a mechanism highly similar to that of its parent protein. Atsttrin, like PGRN, exhibited chondrogenesis-promoting capacity *in vitro* and improved cartilage repair *in vivo*. A model, wherein the generation of a full-thickness defect allowed for the entry of multipotent bone marrow − derived MSCs into the injury area, was used based on the same premise as clinically employed microfracture (Daher et al., 2009; Barry and Murphy, 2013). The stimulation of chondrogenic differentiation by Atsttrin occurred through TNFR1 and TNFR2 signaling, although TNFR2 seemed to be the major mediator of this effect as indicated by the comparative analysis of cartilage regeneration in WT, TNFR1–/–, or TNFR2–/– mice. The differentiating effects of PGRN and Atsttrin were each decisively arbitrated by JunB transcription factor activation, although the activation was achieved through different signaling pathways. Compared with the key role of Erk1/2 signaling in the chondrogenic effect of PGRN, Atsttrin relies solely on Akt signaling. The current results revealed some divergence with previous reports, as the slight activation of Erk1/2 signaling following Atsttrin treatment has been reported in chondrocytes previously [19]. However, the present results largely agree with previous findings. During the progression of arthritis, for example, Atsttrin similarly exhibited its cartilage-protective effect through both inhibiting TNFα/TNFR1-mediated inflammation and activating anabolic TNFR2 pathways [reviewed in (Wei et al., 2016)].

As parts of joints, the synovium and bone also play a non-negligible role in the development and progression of chondral defects. Atsttrin has also exhibited an anti-synovitis effect in both inflammatory and degenerative arthritis mouse models (Tang et al., 2011; Zhao et al., 2015; Wei and Liu, 2018). Recent studies also indicated that therapeutically targeting bone metabolism could mitigate osteoarthritis progression (Chu et al., 2019; Zheng et al., 2020). PGRN, as a downstream molecule of BMP-2, promoted bone formation under physiological and diabetic conditions (Zhao et al., 2013; Wei et al., 2019). A 3D-printed Atsttrin-incorporated alginate/hydroxyapatite scaffold was shown to effectively promote bone defect regeneration (Wang et al., 2015). These findings indicated that Atsttrin might also protect cartilage by reducing synovial inflammation and enhancing the strength of bone. The present study did not analyze bone or synovial tissues. Future studies should assess the potential of Atsttrin to simultaneously address the multidimensional aspects of joint injury and degeneration. Additionally, since Atsttrin was generated based on the binding capacity of PGRN-TNFRs, it was also expected that Atsttrin would lose its activities in TNFR1/2 double-deficient mice, which needs further investigation.

## Conclusion

In conclusion, this study proposed a model for the role of Atsttrin in cartilage repair (Figure 5G). This model illustrated that Atsttrin bound to TNFR2 and activated Akt signaling, followed by JunB activation, resulting in cartilage regeneration. Cumulatively, these findings not only provided new insights into the role of Atsttrin in cartilage homeostasis but also might lead to new therapeutic alternatives for cartilage damage as well as other related joint diseases.

## Data Availability Statement

The original contributions presented in the study are included in the article/Supplementary Material, further inquiries can be directed to the corresponding author.

## Ethics Statement

The animal study was reviewed and approved by the Institutional Animal Care and Use Committee of New York University.

## Author Contributions

JW designed and acquired the data. KW analyzed and interpreted the data and performed the statistical analysis. AH and CL edited the manuscript. All authors drafted and reviewed the manuscript.

## Conflict of Interest

The authors declare that the research was conducted in the absence of any commercial or financial relationships that could be construed as a potential conflict of interest.
