# Supplementary material for: Atsttrin Promotes Cartilage Repair Primarily Through TNFR2-Akt Pathway
Source: Front Cell Dev Biol. 2020 Oct 29;8:577572. doi: 10.3389/fcell.2020.577572 (PMC7658268; doi:10.3389/fcell.2020.577572)
Supplement: Supplementary file 1 [file Table_1.DOCX]

**

Supplementary Fig. 1. Lower magnification image of Figure 3 showing that Atsttrin accelerated cartilage repair through TNFRs.** The defected areas shown in Figure 3 are indicated.
